# Analysing human population movement data for malaria control and elimination

**DOI:** 10.1186/s12936-021-03828-6

**Published:** 2021-06-30

**Authors:** Greta Tam, Benjamin J. Cowling, Richard J. Maude

**Affiliations:** 1 WHO Collaborating Centre for Infectious Disease Epidemiology and Control, School of Public Health, Li Ka Shing, Faculty of Medicine, The University of Hong Kong, Hong Kong, China; 2 Mahidol-Oxford Tropical Medicine Research Unit, Faculty of Tropical Medicine, Mahidol University, Bangkok, 10400 Thailand; 3 Centre for Tropical Medicine and Global Health, Nuffield Department of Medicine, University of Oxford, Oxford, OX3 7LG UK; 4 The Open University, Milton Keynes, MK7 6AA UK; 5 Harvard TH Chan School of Public Health, Harvard University, Boston, MA 02115 USA

**Keywords:** Malaria, Human population movement, Human mobility, Travel

## Abstract

**Background:**

Human population movement poses a major obstacle to malaria control and elimination. With recent technological advances, a wide variety of data sources and analytical methods have been used to quantify human population movement (HPM) relevant to control and elimination of malaria.

**Methods:**

The relevant literature and selected studies that had policy implications that could help to design or target malaria control and elimination interventions were reviewed. These studies were categorized according to spatiotemporal scales of human mobility and the main method of analysis.

**Results:**

Evidence gaps exist for tracking routine cross-border HPM and HPM at a regional scale. Few studies accounted for seasonality. Out of twenty included studies, two studies which tracked daily neighbourhood HPM used descriptive analyses as the main method, while the remaining studies used statistical analyses or mathematical modelling.

**Conclusion:**

Although studies quantified varying types of human population movement covering different spatial and temporal scales, methodological gaps remain that warrant further studies related to malaria control and elimination.

## Background

In the past 20 years, there has been significant progress in malaria control due to new technologies and increased political and financial commitment globally. Since 2000, 20 countries no longer have endemic malaria [1], annual global malaria incidence has decreased by 36% and mortality has decreased by 60% [2, 3]. Currently, nearly half of the world has eliminated malaria [4] and eradication is envisioned [5]. The last attempt at eradication failed 50 years ago, with human population movement (HPM) cited as one of the major reasons [6].

The World Health Organization (WHO) has declared that all countries should aim for malaria elimination as their ultimate goal, regardless of their malaria burden. Subnational elimination is advocated as a preliminary step for large countries when certain areas of the country have interrupted local transmission. As most countries have diverse transmission intensity, elimination would require stratifying a national map by receptivity and transmission characteristics for targeted malaria interventions [7]. Receptivity is the ability of an ecosystem to transmit malaria, thus determining local transmission intensity. In a non-receptive area, vulnerability (the risk posed by imported infections) would be the major concern [8]. A receptive area would be further stratified according to whether there was transmission. An area with transmission would be stratified according to whether there was a focus or transmission was diffuse. Areas with diffuse transmission would be stratified according to the degree of transmission, while foci would be stratified by whether there was ongoing, interrupted or no local transmission [7].

In order to accurately track transmission within and between stratified areas, increasingly granular surveillance is needed [7]. In areas of very low transmission, surveillance should detect foci at the scale of individual villages or health facility catchment areas. Meanwhile, countries with high transmission usually stratify their subnational areas according to districts or provinces [9, 10]. Countries require detection and response to intra and international transmission on an increasingly fine scale as countries approach elimination [11]. Surveillance systems could, therefore, capture HPM of infected migrants as well as HPM of the malaria infected local population to quickly identify potential importation and travel-related hotspots. However, routine collection of such data as part of malaria surveillance is often not done or is limited in scope. A wide variety of alternative data sources and analytical methods have been used to quantify HPM relevant to malaria control and elimination covering different spatial and temporal scales and each with their own strengths and limitations.

This review categorizes and describes studies of HPM patterns relevant for malaria control and elimination, including the sources of data used, as well as methods for combining and analysing different datasets, to quantify varying types of HPM and identify methodological gaps for future investigation.

## Methods

### Paper inclusion criteria

Studies were identified by literature search in PubMed, Ovid and Google Scholar. The combination of keywords “human mobility/mapping/movement/travel” and “malaria” was used. The most highly cited and relevant literature in the past 10 years were included and reference lists of related papers were looked at.

### Categorizing studies of human population movement relevant to malaria control and elimination

Studies were deemed relevant if their findings had policy implications that could help to design or target interventions for malaria control and elimination. A framework for categorizing HPM according to spatial and temporal scales was described by Stoddard et al*.* [12]. Here, this framework was adapted and extended by identifying studies of HPM relevant for malaria control and elimination. Plotted on the spatial and temporal scales of human mobility, studies roughly fell into a few broad categories: neighbourhood daily and seasonal HPM, regional periodic HPM, intra-national periodic HPM, international periodic HPM, international seasonal HPM, international migration HPM (Table 1). The studies were categorized by their main method of analysis.

**Table 1:** Literature review data categorized* according to spatiotemporal scales of human mobility

	Spatial scales of human mobility			
	Neighbourhood	Regional	Intranational	International
Temporal scales of human mobility				
Daily	Carrasco-Escobar et al. [13] Hast et al. [14] Searle et al. [15]			
Periodic	Carrasco-Escobar et al. [13] Hast et al. [14] Searle et al. [15]	Knudson et al. 2020 [16]	Sinha et al. 2020 [17], Guerra et al. 2019 [18], Chang et al. 2018 [19], Ihantamalala et al. 2018 [20], Tatem et al. 2014 [21], Wesolowski et al. 2014 [22], Wesolowski et al. 2012 [23], Le Menach et al. 2011 [24]	Tessema, Wesolowski et al. 2019 [25], Tatem et al. 2017 [26], Ruktanonchai et al. 2016 [27], Huang et al. 2013 [28]
Seasonal	Hast et al. [14] Searle et al. [15]		Cohen et al. 2013 [29]	Saita et al. 2019 [30]
Migration				Pindolia et al. 2014 [31], Pindolia et al. 2013 [32], Tatem et al. 2010 [33]

^*^Definition of spatial and temporal categories [34]:

Spatial categories

Spatial categories were defined according to the spatial resolution of sampling

Neighbourhood: Sampling occurred within a district

Regional: Sampling occurred within an administrative division of a country

Intranational: Sampling occurred within a nation

International: Sampling occurred in two or more nations

Temporal categories

Temporal categories were defined according to the temporal resolution of sampling

Daily: The timing of the usual activities of participants’ days was captured

Periodic: Intermittent movement at intervals was captured. In this paper, these intervals are defined as longer than a day [e.g. weekly, monthly], but not capturing seasonal variations

Seasonal: Movement relating to certain seasons of the year was captured, thus capturing seasonal variations of movement

Migration: Movement from one’s place of abode to settle in another country

### Neighbourhood daily and seasonal HPM

Neighbourhood daily and seasonal HPM is measured at the finest spatial and temporal scales. This is important as heterogeneity in exposure can greatly affect individual risk of infection in a local setting where sustained transmission occurs. Tracking movements of susceptible hosts to high risk locations, duration spent in each location, time of day that movement occurred and routes taken can provide insight into individual risk of infection. Observation for at least 2 weeks could give insights into routines during the work week and overall transmission patterns [12].

In recent years, GPS data-loggers and GeoODK (an open-source mobile mapping tool) have been used to collect high-resolution data on neighbourhood daily HPM in areas with malaria transmission [13–15]. These devices may be the best option for tracking HPM in rural areas where cell phone coverage is not universal, although dense tree coverage can reduce the accuracy of GPS data-loggers [12, 35–37]. GPS data-loggers and GeoODK have been used to track HPM in agrarian [13, 14] and riverine [15] populations in malaria transmission settings. These devices tracked small-scale movement, with the furthest trips limited to 200 km. HPM were tracked for up to a year with samples of less than 100 people.

Concurrent travel surveys, malaria risk maps and collection of blood samples have provided important supplementary data [13–15]. GPS has lower indoor accuracy and, therefore, may not accurately show whether an individual was inside or outside their household. A combination of objective and recall methods will enable more accurate qualification of movements with regard to disease risk [12].

Studies using GPS data-loggers or GeoODK in areas with malaria transmission did not rely solely on the device for their sources of data. In combination with malaria risk maps, Searle et al. generated density maps. Together with activity space plots, they were able to analyse seasonal movement patterns and conclude that there was limited movement during the rainy season, but increased long-distance travel during the dry season in rural southern Zambia [15]. Carrasco-Escobar et al*.* generated heatmaps of transit from GeoODK data which showed areas of intensive travel transit and high-connectivity units which were occupation-related in  the rural Peruvian Amazon. This information was supplemented by a travel survey which provided demographic information and reasons for travel [13]. Hast et al*.* [14] also supplemented their GPS data with a travel survey which included coverage of malaria interventions. Malaria testing of participants and seasonal malaria risk maps provided further information. In addition to activity space plots, movement intensity plots were generated. While Searle et al*.* and Carrasco-Escobar et al*.* [13, 15] carried out descriptive analyses, Hast et al*.* added statistical analyses (Table 2). Malaria risk scores were calculated using malaria risk maps and the time an individual spent at a location. Association of movement patterns with demographics, malaria incidence and malaria risk were also calculated. These concluded that there was a diverse mobility pattern along the banks of Lake Mweru in northern Zambia and that no significant associations were found [14].

**Table 2:** Literature review data categorized by main method of analysis

Main method of analysis	Publication	Details
Descriptive	Carrasco-Escobar et al. [13]	Heatmap of transit
	Searle et al. [15]	Density maps, activity space plots
Statistical	Knudson et al. [16]	Topological data analysis
	Sinha et al. [17]	Logistic regression
	Hast et al. [14]	Movement intensity plots, activity space plots, malaria risk score, chi square test, Wilcoxon rank sum test
	Saita et al. [30]	Logistic regression
	Tatem et al. [26]	Network community detection
	Ruktanonchai et al. [27]	Logistic regression
	Pindolia et al. [31]	Hot spot analysis, linear regression
	Tatem et al. [21]	Regression trees, network analysis
	Cohen et al. [29]	Logistic regression, regression trees
	Huang et al. [28]	Network analysis
	Pindolia et al. [32]	Network analysis
	Tatem et al. [33]	Network analysis
Mathematical model		
Individual-based	Tessema, Wesolowski et al. [25]	
	Ihantamalala et al. [20]	
	Wesolowski et al. [22]	
	Wesolowski et al. [23]	
	Le Menach et al. [24]	
Compartmental	Chang et al. [19]	
	Guerra et al. [18]	
	Pindolia et al. [31]	
	Le Menach et al. [24]	

### Regional and intranational periodic and seasonal HPM

Regional periodic HPM is measured at fine spatial resolution units to detect variations at the micro-epidemiological level, such as variation in malaria risk between villages in an endemic region [38–40]. Micro-epidemiological variations in malaria exposure become more apparent in low and moderate transmission settings because heterogeneity is no longer obscured when a proportion of the population remains malaria free for years, while small groups of households experience multiple episodes [41–43]. The area where this cluster of higher-than-average malaria prevalence occurs is spatially defined as a hotspot [44].

Genetic epidemiologic data can help to define regions into spatial units for targeted interventions, identify sources and sinks and reconstruct the transmission chain. Genetic data such as single nucleotide polymorphisms (SNP), short haplotypes, microsatellites and whole genomes can be used to derive genetic measures of parasite relatedness. Meanwhile, epidemiological data regarding clinical cases, population prevalence and history of travel can provide a measure of HPM. Genetic data tends to underestimate parasite relatedness, while epidemiological data tends to overestimate HPM connectivity [45]. Therefore, a combination of genetic and epidemiological data may provide a more accurate map of malaria transmission [45, 46].

Knudson et al. demonstrated the use of genetic epidemiologic data to define a spatial unit, which was the area that contributed 95% of diagnosed malaria cases in a catchment facility in a town on the Columbian Pacific Coast. A combination of passive and active surveillance was used: passive surveillance at local clinics included microsatellite and SNP genotyping of positive samples and carrying out travel surveys, while active surveillance included testing for asymptomatic malaria in households. Using the surveillance data, epidemic curves could show not only the temporal distribution of cases in the spatial unit by month, but also the regions of origins. Topological data analysis generated a network representation of parasite populations connectivity underlying the epidemic curve. This network was overlaid onto a map to show the spatial connectivity of cases in the spatial unit. As all three parasite populations were found in the town of Guapi, this was deemed a sink for cases imported from the surrounding rural areas in the spatial unit [16].

A variety of methods have been used to collect data on intra-national periodic and seasonal HPM. Travel surveys ranging from a sample size of less than 3000 to national surveys have often been used [17–19, 22, 29]. These were combined with national malaria incidence or prevalence data [17, 18, 20, 22, 29]. Travel surveys were also combined with mobile phone call data records (CDRs) and genetic data, thereby applying genetic epidemiologic data at the national level [19]. The use of mobile phone CDRs to measure intra-national HPM has become increasingly widespread as mobile phone penetration increased globally, particularly in low-income countries. This big data approach allows the direct measurement of individual-level HPM between regions on a population scale. Mobile phone CDRs are stored by their mobile phone operators. Mobile phone activity such as calls, texts, top-ups and sending money are logged as digital data points which stores information on the SIM card used and the location of the nearest cell phone tower [47–49]. Mobile phone CDRs were combined with malaria prevalence data [20–23] and traffic data [24]. Most studies used mathematical models to analyse the combined data, with the exception of three studies [17, 21, 29].

Cohen et al*.* [29] classified cases as locally acquired or imported, according to travel history. A logistic regression mixed model was then used to predict whether an imported case was associated with a locally acquired case. Regression trees were used to create case-based malaria risk maps, a method which was also used by Tatem et al*.* [21]. Using network analysis, Tatem et al*.* [21] combined the predicted risk with connectivity (derived from mobile phone CDRs), to quantify overall risk flow. Sinha et al*.* [17] used a simple empirical method to classify sources and sinks by calculating the amount of travel to a destination or from an origin, weighted by the number of enrolled cases resident in the origin subdistrict and the Annual Parasite Index (API). Descriptive analysis and logistic regression were used to determine which groups of people with malaria were travelling, where they were travelling and why [17].

Sinha et al.’s study [17] served as a complementary analysis to Chang et al.’s study which used a compartmental model combining genetic data, travel surveys and mobile phone CDRs to distinguish areas of high malaria transmission and frequent importation [19]. Two other studies used compartmental models to estimate transmission and importation [18, 24]. Four studies used individual-based models to identify routes of parasite importation [20, 22–24]. Ihantamalala et al*.* [20] compared two methods, using different sources of data in combination with mobile phone CDRs: one model used prevalence estimates from the Malaria Atlas Project, while another used nationally reported cases. Both methods reached similar conclusions [20]. Wesolowski et al*.* [23] used mobile phone CDRs and estimates from a malaria prevalence map in their model. Subsequently, they estimated the ratio of monthly imported-to-clinical cases in Nairobi by comparing their predicted imported cases to malaria incidence derived from cross-sectional clinical surveys, thus identifying transmission foci in a low-risk urban setting [23]. In a later study, Wesolowski et al*.* [22] compared two methods, using different sources of data in combination with prevalence estimates from the Malaria Atlas Project. One model used travel survey data, while another used mobile phone CDRs. Both methods reached similar conclusions regarding estimated malaria importation, although the volume of exchange was smaller for the model using travel survey data [22].

### International periodic and seasonal HPM

In addition to using travel surveys [17, 25, 30], genetic data [25] and mobile phone CDRs [25, 27], to collect data on international periodic and seasonal HPM, flight data [28], nationally reported statistics on imported malaria [26] and census data [27] have also been used.

Only one study used a mathematical model: Tessema et al*.* used an individual-based model combining genetic data, travel surveys and mobile phone CDRs to track cross-border malaria transmission [25]. Saita et al*.* used logistic regression to analyse seasonal HPM and behavioural patterns in malaria hotspots on the border [30]. Ruktanonchai et al*.* compared HPM patterns between census-based migration data and mobile phone CDRs. They concluded that similar to mobile phone CDRs, migration data could predict short-term movement patterns [27]. Two studies examined global malaria connectivity through air travel. Using network analysis, Huang et al*.* combined prevalence maps from the Malaria Atlas Project with flight data [28]. Tatem et al*.* used national imported malaria statistics and performed network community detection to map the importation of malaria to non-endemic countries [26].

### International migration HPM

Census and malaria prevalence data were commonly combined [31–33], together with travel surveys [32], to analyse international migration HPM. Two studies used network analysis: Tatem et al*.* mapped countries that formed communities connected by high levels of infection movement [33]. With the addition of travel survey data to census and prevalence data, Pindolia et al*.* developed demographically-stratified estimates of HPM and malaria movement [32]. In another study, Pindolia et al*.* used hot spot analysis to map origin-specific immigrant hotspots in destination countries. Linear regression was used to model migration, and a compartmental-based model, developed in previous studies [8, 24], was used to estimate malaria importation propensity [31].

## Discussion

Most studies combined data to provide a more comprehensive picture of HPM patterns [50]. In addition, some studies demonstrated that different data sources could complement each other [20, 22, 27]. However, evidence gaps along the spatiotemporal scales need to be filled with future studies.

All studies tracking neighborhood HPM included GPS as a data source, which could track not only daily HPM, but also seasonal HPM [13–15]. Evidence gaps exist for daily HPM beyond the neighborhood (Table 1). Tracking daily HPM is measurement at the finest temporal scale. Routine activities in a participant’s day would usually be confined to a relatively short distance. Sampling on a larger scale (e.g. regional or intra-national) would be expensive [12] and unlikely to detect significant patterns due to the lack of overlap in participants’ areas of travel and a small sample size. However, detecting daily cross-border movements (routine contiguous international HPM) might be feasible. Despite the frequency and large volume of contiguous international HPM travel, there is a lack of detailed HPM assessment in this aspect, which would have a significant impact on malaria elimination [8].

All studies capturing neighbourhood HPM used GPS data-loggers and GeoODK [13–15]. Although this captured movement at the finest scale, sampling and analysis were limited by this method of data collection. Studies were limited by a small sample size with non-probability sampling. As GPS data-loggers and GeoODK are highly subject to user error, children were excluded, limiting the representativeness of the population in the neighbourhood. Due to lower indoor accuracy of the devices, participants may be inaccurately classified as indoor or outdoor, thus affecting the accuracy of activity space-plots. Data analysis was mostly limited to descriptive methods, despite the addition of malaria risk maps and travel surveys [13, 15]. Hast et al*.* [14] used statistical methods which highlighted HPM patterns among demographic groups. However, the lack of significant associations with incident parasitaemia may have been due to the limited power of the study. In high transmission settings, saturation of malaria risk may result in movement patterns not significantly predicting individual risk.

Evidence gaps exist for HPM at the regional scale, with only one study in this category, which measured periodic HPM (Table 1). Regional HPM patterns might be overlooked as using GPS would be unfeasible, yet HPM still needs to be measured at fine spatial resolution units to detect micro-epidemiological changes. Genetic epidemiologic data used to track regional periodic HPM could be analysed to detect whether any seasonality and trends existed, to build a more accurate temporal picture and fill the existing data gap.

To capture regional HPM, Knudson et al*.* [16] used genetic epidemiology to define a malaria transmission unit. This method may be suitable for areas with low to moderate malaria transmission, where hotspots become apparent. In high transmission areas, genetic epidemiology data might instead be used to evaluate interventions by analysing genetic correlates of declining transmission [45]. In addition to defining a malaria transmission unit, Knudson et al. [16] used genetic epidemiology to estimate the size of the asymptomatic reservoir and provide information on parasite genetics related to drug resistance and false negatives from rapid diagnostic tests. However, the use of molecular surveillance is still at an early stage. More studies are needed with larger sample sizes in different transmission settings, to decrease bias and explore how genetic and epidemiological data can best be combined to accurately track HPM.

Surveys were a commonly used source of data to capture intra-national and international HPM. Surveys provide important information on reasons for travel and for identifying hotpops (demographic groups with higher-than-average malaria prevalence) [8, 51]. However, surveys are generally cross-sectional, prone to recall bias, may lack detail and are difficult to conduct on a large-scale, thus they are also prone to sampling bias. Sampling malaria patients from healthcare facilities may be biased by differential access to healthcare [17, 29], while community surveys may be biased by the lack of working men and visitors who are active acquirers of infection [18, 22]. Qualitative studies could be used to increase the granularity of survey data in a few ways: they could provide some details lacking in surveys, purposefully sample populations lacking access to healthcare and explore the reasons why, as well as overcome recall bias using diary studies, for example.

Despite the lack of routine HPM data, Guerra et al. [18] used annual Malaria Indicator Survey [MIS] data, while Cohen et al*.* [29] used survey data from the Swaziland National Malaria Control Programme. Guerra et al*.* [18] identified hotpops and estimated importation and residual transmission. The analysis was limited by the sampling and spatial resolution of the survey. As only residents were sampled, importation rates only considered passive acquirers of infection [returning residents]. As MIS did not record the off-island destinations of travel, it was not possible to map the exact sources of malaria transmission. The case-based risk maps generated by Cohen et al. [29] and Tatem et al*.* [21] were limited by small sample sizes, as only one year of data was used. Nevertheless, Cohen et al*.* [29] was the only intra-national study that took seasonality into account. Sinha et al*.* [17] mapped sources and sinks, hotpops and HPM patterns from survey and incidence data, which were easily collectable. The analysis was limited by the spatial resolution of the geographic data, which was at the union level instead of the village level.

Most intra-national studies included mobile phone CDRs in their analyses. Unlike surveys, mobile phone CDRs have large sample sizes. However, data is limited by cell phone tower density and sampling may be biased, as subscribers are more likely to be educated, urban and male [52]. In addition, it cannot directly identify hotpops and cannot track cross-border HPM without tracking handset IDs and combining data from multiple countries. Despite the differences between surveys and mobile phone CDRs, Wesolowski et al*.* [22] concluded that both sources could quantify broad travel patterns, including regional differences. Used together, they could potentially complement each other to form a detailed picture of HPM. In addition to surveys and mobile phone CDRs, Chang et al*.* [19] used genetic epidemiology data. This was the only intra-national study that used genetic epidemiology data. Their genetic mixing index was not biased by incidence underestimation and was used in a transmission setting and geography where commonly used methods could not easily distinguish genetic differentiation. However, it was limited by a small sample size. Unlike Knudson et al*.* [16], only passive surveillance was used, therefore asymptomatic and subclinical infections were not sampled and results may not have been representative of the entire parasite population.

A few studies used prevalence estimates from the Malaria Atlas Project. These were limited by the lack of seasonality in the estimates [18, 20, 22]. However, incidence data also had limitations. Incidence data from nationally reported cases to health facilities may be biased by accessibility to healthcare and representative of symptomatic cases only. In endemic areas, there may be increased immunity, leading to fewer symptomatic cases. In addition, cases were aggregated per month, which could influence the accuracy of the mathematical model [20].

Only one study included traffic data. Le Menach et al*.* [24] included ferry traffic data, in an attempt to more accurately capture travel between Zanzibar and the mainland. Despite the inclusion, it could not fully account for informal movements via small fishing boats.

Two studies tracking international HPM included mobile phone CDRs. Tessema et al*.* [25] used mobile phone CDRs to supplement survey and genetic data. This was the only study tracking international HPM that included genetic data. Ruktanonchai et al*.* [27] used mobile phone CDRs to compare how well census-derived migratory data predicted short-term HPM and found that HPM movement patterns were strongly correlated. Therefore, census-derived migratory data was used to predict HPM patterns across Meso-America. However, as the data did not record individual-level risk, inclusion of travel history surveys might have complemented the analysis.

Two studies tracked HPM through air travel. Tatem et al*.* [26] used routine data from nationally reported statistics on imported malaria. However, the data likely represented only one-sixth of all imported cases globally. In addition, due to global differences in health systems, there would be heterogeneities in case reporting. Mixed species infections might have obscured certain species and data was pooled across a decade, in order to have sufficient data. Nevertheless, the meta-analysis detected a small number of high-traffic routes that accounted for 56% of imported malaria to non-endemic countries and the occurrence of strong spatial clustering of Plasmodium species, which could inform global malaria policy. Huang et al*.* [28] tracked passenger flows weighted by malaria prevalence and highlighted risk routes for artemisinin resistance spread in Southeast Asia using Malaria Atlas Project prevalence maps and flight schedules. However, this was limited by the lack of data on individual-level risk and the reliance on travel data when examining artemisinin resistance, thus heterogeneity in resistance throughout the region was not accounted for. Amongst studies tracking international HPM, only Saita et al*.* [30] accounted for seasonality.

All studies tracking migration used census data [31–33]. However, census data did not provide fine-scale HPM data and may have missed highly mobile populations that could contribute to malaria transmission.

## Conclusion

Evidence gaps exist in tracking routine cross-border HPM and HPM at a regional scale. Only a few studies accounted for seasonality, despite the importance in malaria transmission. A wide variety of data sources and methods were used to analyse HPM data for malaria control. The advantages and limitations of each one should be considered carefully, to enable different data sources to complement each other and build an accurate spatio-temporal picture for malaria control. For large-scale collection of HPM data outside of research settings, especially in lower and lower middle income countries, the additional cost and resource requirements should be addressed.

## Data Availability

All data are from published studies.
